# Eco-Friendly and Efficient Extraction of Polysaccharides from *Acanthopanax senticosus* by Ultrasound-Assisted Deep Eutectic Solvent

**DOI:** 10.3390/molecules29050942

**Published:** 2024-02-21

**Authors:** Jiaojiao Xue, Jianqing Su, Xueyan Wang, Rui Zhang, Xiaoli Li, Ying Li, Yi Ding, Xiuling Chu

**Affiliations:** College of Agronomy and Agricultural Engineering, Liaocheng University, Liaocheng 252000, China; x15065441211@163.com (J.X.); wangxueyan202203@163.com (X.W.); 17863709708@163.com (R.Z.); lxl15006995983@163.com (X.L.); ly15963373832@163.com (Y.L.); djy15373023047@163.com (Y.D.)

**Keywords:** deep eutectic solvents, extraction, *Acanthopanax senticosus* polysaccharide

## Abstract

A green extraction method was developed using deep eutectic solvent extraction for the polysaccharide from *Acanthopanax senticosus* (*A. senticosus*). Among the eight types of DES prepared, the DES with a ratio of 1:4 L-malic acid to L-proline was found to be a suitable extraction solvent based on the extraction efficiency. The extraction parameters were optimized by Plackett–Burman and response surface methodology (RSM). The best extraction conditions were found for L-malic acid. Under the conditions of an L-malic acid/L-proline ratio of 1:4, ultrasonic power of 240 W, material–liquid ratio of 31.068 g/mL, water content of 32.364%, extraction time of 129.119 min, and extraction temperature of 60 °C, the extraction rate of *A. senticosus* polysaccharides was 35.452 ± 0.388 mg-g^−1^. This rate was higher than that of polysaccharides obtained by hot water extraction (13.652 ± 0.09 mg-g^−1^). The experimental results were best fitted by the quasi-secondary kinetic model when compared to two other kinetic models. Electron microscopic observations showed that DESs were more destructive to plant cells. The polysaccharide extracted from DESs had more monosaccharide components, a lower molecular weight, a higher antioxidant capacity, and superior anti-glycation activity compared to polysaccharides extracted from water (ASPS-PW). This study demonstrates the effectiveness of DESs in obtaining polysaccharides from *A. senticosus*.

## 1. Introduction

*Acanthopanax senticosus* (*A. senticosus*), belonging to the family Araliaceae, genus *Acanthopanax*, is widely distributed throughout northern Asia, especially in northeastern China, the Russian Far East, Japan, and Korea [1]. *A. senticosus* is used as an analogy with ginseng, also known as Siberian ginseng, a famous traditional Chinese herb, which is rich in polysaccharides, polyphenols, flavonoids and saponins, and other active ingredients, and, therefore, has very positive pharmacological effects, mainly in enhancing the immunity and antioxidant capacity of animal organisms [2,3,4], anti-stress [5], protecting the nervous system and cardiac muscle cells [6], enhancing anti-inflammatory and antibacterial effects [7,8], and hypoglycemic effects [9].

Polysaccharides are large organic molecules commonly found in living organisms and are active macromolecules indispensable in maintaining the everyday life activities of organisms. Polysaccharides have immune-modulating, antioxidant, antiviral, anti-inflammatory, and antitumor effects [10,11,12,13,14,15]. Polysaccharides extracted from *A. senticosus* have been found to have various pharmacological activities such as immunomodulatory [16], antioxidant [17], anti-inflammatory [18], and antitumor activities [1] according to the reported literature. Traditional polysaccharide extraction methods mainly include water extraction, enzyme extraction, and alkali extraction. Hot water extraction is the most commonly used method to extract polysaccharides. The main advantage is that the operation method is simple and low-cost, but the time consumed is long and the extraction temperature is high, which not only leads to low purity of the extracted polysaccharides but also, to a certain extent, the long time spent on high-temperature extraction consumes high energy. Although the acid–base extraction method can improve the extraction rate of polysaccharides, it is easy to destroy the structure of polysaccharides, reduce the activity of polysaccharides, and the method is not green [19]; Enzyme-assisted extraction can improve the rate of polysaccharides to a certain extent, but the effect of the extraction is dependent on the type of enzyme and activity. In addition, the addition of enzymes increases the burden of polysaccharide purification and limits the preparation of high-quality polysaccharides [20]. Therefore, developing a green and efficient method to extract polysaccharides is important.

Professor Abbott first developed the idea of deep eutectic solvents (DESs) in 2001 and introduced it in 2003 [21,22], Choi et al. (2011) came up with the idea of “natural deep eutectic solvents” (NADES). Deep eutectic solvents come from primary metabolites like sugars, amino acids, organic acids, polyols, and choline derivatives [23,24]; in addition, water is also a part of the composition of naturally occurring low eutectic solvents, and the addition of water can reduce the viscosity and hydrogen-bonding structure of NADES, taking into account the effectiveness and economic effects of extraction. Addition reduces the viscosity and hydrogen-bonding structure of NADES, and the addition of appropriate amounts of water to NADES is encouraged considering the effectiveness and economic effect of the extraction. NADES is often a liquid substance formed by a hydroxyl bond acceptor (HBA, some of the salts such as the quaternary ones) and a hydrogen bond donor (HBD, such as the polyols, urea, and the carboxylic ones) [25,26]. The components of NADES are not only capable of joining each other through hydrogen bonds but also of providing or accepting external electrons or protons to form hydrogen bonds [27], the latter allowing them to solubilize a wide variety of substances, including proteins, amino acids, drugs, and polysaccharides [28], among others. Therefore, they can effectively extract polar or non-polar components from plants. They have been widely used to extract natural active substances from plants, such as polysaccharides [29], flavonoids [30,31], phenols [32], and anthocyanins [33,34]. They are more effective than traditional extraction solvents for extracting natural active molecules. The use of deep eutectic solvents as extraction solvents has the benefits of higher efficiency, lower time, reduced cost, non-toxicity and biodegradability, and improved product purity [35]. Studies exploring different types of deep eutectic solvents for extracting active ingredients in traditional Chinese medicine have become a favored direction. More and more studies have shown that deep eutectic solvents have good biodegradability and biocompatibility and are expected to be an excellent green alternative to organic solvents for the extraction of natural products. Scientific research has shown that using ultrasound can cause an “acoustic perforation” in the membranes of plant cells. This lets bioactive substances that were previously inside the cells escape [36,37]. The application of ultrasound has been found to enhance the swelling index of the plant tissue matrix, thereby facilitating the desorption and diffusion of solutes. This phenomenon ultimately results in an elevated extraction rate [38]. The utilization of ultrasound-assisted deep eutectic extraction for extracting active compounds from plants has emerged as a prominent area of research, as depicted in Table 1. Response surface methodology (RSM) is a statistical technique utilized to identify the most favorable conditions for a process response variable. It also enables the assessment of the relationship between a series of controlled trials and the observed outcomes of one or more chosen variables [39,40,41]. Additionally, this method serves as an optimization technique that examines the interrelationships among multiple factors, thereby assessing and predicting the collective impacts resulting from these variables.

We present in this study an ultrasound-assisted method for the extraction of ASPS by DESs, and for the first time, a deep eutectic solvent of proline–malic acid was used for the extraction of polysaccharides from *A. senticosus*. This study aimed to assess the practicality and effectiveness of eight distinct DESs for extracting *A. senticosus* polysaccharides. The researchers improved the extraction conditions and looked at the kinetics using the response surface methodology (RSM) and the Plackett–Burman (PB) and Box–Behnken design (BBD) methods. Furthermore, the utilization of alcohol precipitation was employed to acquire polysaccharides from the extracts. The potential for reusing this deep eutectic solvent was investigated, and the polysaccharides obtained were subjected to analysis for their monosaccharide composition, molecular weights, anti-saccharides, and antioxidant activities. This method lays a substantial rational foundation for exploring the use of DESs to extract polysaccharides from *A. senticosus* in an efficient and rapid manner.

## 2. Results and Discussion

### 2.1. Effect of Different DESs on the Yield of ASPS

**Figure 1:**
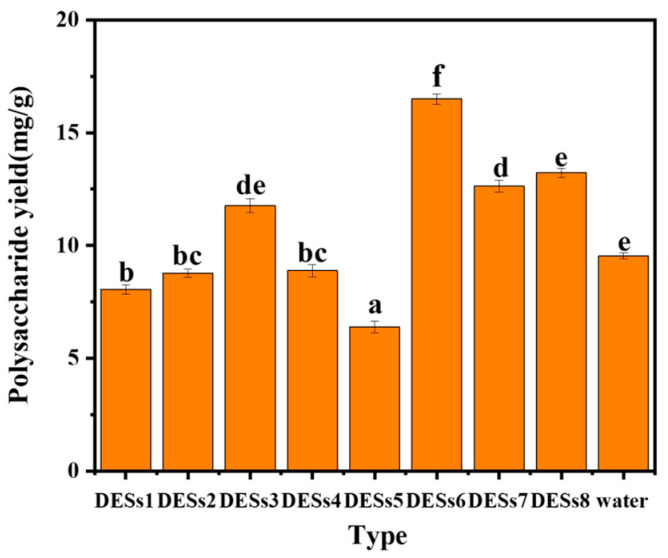
Extraction of polysaccharide content with different extractants. Note: Different letters above the columns represent significant differences (*p* < 0.05).

DESs consist of hydrogen bond acceptors in a particular ratio with donors. Physicochemical properties of DESs include solubility, viscosity, and density, among which polarity influences the solubility of polysaccharides. Referring to several articles and considering the polarity and solubility of polysaccharides, eight DESs were selected (described in Section 2.2). The results are shown in Figure 1: DES6 showed the best extraction effect which was higher than that of water under the same conditions, while DES7 and DES6 showed the worst extraction effect. This may be related to the polarity of DESs, and DES6 degrades lignin and cellulose [48], which also enhances its effect on the solubilization of polysaccharides. Therefore, DES6 was chosen for the following experiments.

### 2.2. Selection of Extraction Methods

**Figure 2:**
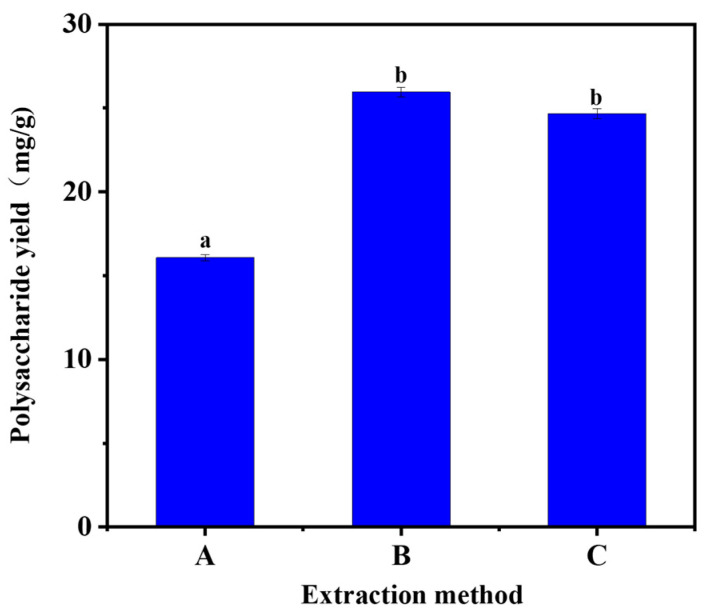
Selection of extraction methods. (A: water bath, B: ultrasonic, C: shaking bed). Note: different letters above the columns represent significant differences (*p* < 0.05).

As shown in Figure 2, it can be seen that shaking-bed and ultrasonic assistance were not significant for the extraction of Acanthopanax polysaccharides. However, both yields were higher than that of hot water extraction. The possible reason for this is that the ultrasound-assisted method is destructive to the cell wall of *A. senticosus*; the shaking bed is less destructive, but it promotes the whole mixing of *A. senticosus* powder and the extraction solution through constant shaking. However, compared with the ultrasound-assisted method, the upper-temperature limit of the shaking bed was smaller, which had some limitations for investigating the effect of temperature on the extraction of *A. senticosus* polysaccharides by DESs, so the ultrasonic extraction method was chosen.

### 2.3. Infrared Spectroscopy of DESs

**Figure 3:**
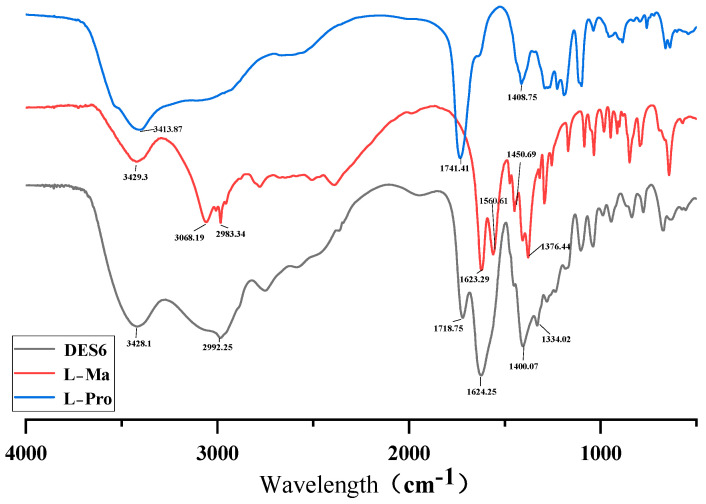
FT-IR spectra (DES6, L-Ma, and L-Pro).

In this experiment, DESs formed by the ratio of L-proline and L-malic acid were selected to extract the polysaccharide compounds. The low eutectic formation cannot be separated from the hydrogen-bonding connection between the mixtures. Comparison of FT-IR spectra of L-proline, L-malic acid, and DES6 (L-Pro–L-Ma) clearly demonstrated the overlapping spectra of L-proline, L-malic acid, and DESs as well as the predominance of DES6. FT-IR (Fourier-transform infrared) spectroscopy is a molecular vibrational spectroscopy method in which the most analytical value is between the frequencies 4000–1300 cm^−1^ (the functional group region), as shown in Figure 3. The FT-IR spectra of DES6 showed that the OH bands of L-Pro and L-Ma overlapped up to 3428.1 cm^−1^ and a strong broad peak at 3685–3277 cm^−1^, and the intrinsic frequency of a hydroxyl group appeared at 3700–3200 cm^−1^, indicating the formation of a large number of hydrogen bonds in DES6. From the figure, it can be observed that the infrared spectrum of DES has a broad peak at 3270–2823 cm^−1^ and L-malic acid has two very narrow peaks at the same wavelength that L-proline has no peaks, reflecting the fact that DES6 fitted the advantages of the two and also providing supporting evidence for the experiments. The presence of these two functional groups in DES6 was also indicated based on the intrinsic frequency of the appearance of C=C and C=O in the spectrum. The absorption peak of L-proline at 1741 cm^−1^ and the two absorption peaks of L-malic acid at 1623–1560 cm^−1^ were fitted to the two large absorption peaks of the DES between 1718–1624 cm^−1^ absorption peaks. That is, at 2761–1497 cm^−1^, the DES had two distinct absorption peaks, which were broader and more pronounced than the absorption peaks of L-Pro and L-Ma, which strengthened the role of the functional groups of the two, and showed the unsaturated bonding and the distinctive characteristics of N=H. The FT-IR spectra of L-Pro and L-Ma showed the absorption peaks at 1408 cm^−1^ and the absorption peaks at 1376 cm^−1^ shifted to 1400 cm^−1^ and 1334 cm^−1^ for DES6, which is closely related to the stretching vibration for single bonds (C–C, C–O, and C–N, etc.). These results theoretically support the efficient extraction of goal ingredients by the Pro–Ma DES. Moreover, the influence of CO_2_ on the results of IR spectroscopy was no longer considered because the CO_2_ content of all samples was deducted from the CO_2_ background during FT-IR spectroscopy.

### 2.4. The Effect of Molar Ratio on the Yield of ASPS

**Figure 4:**
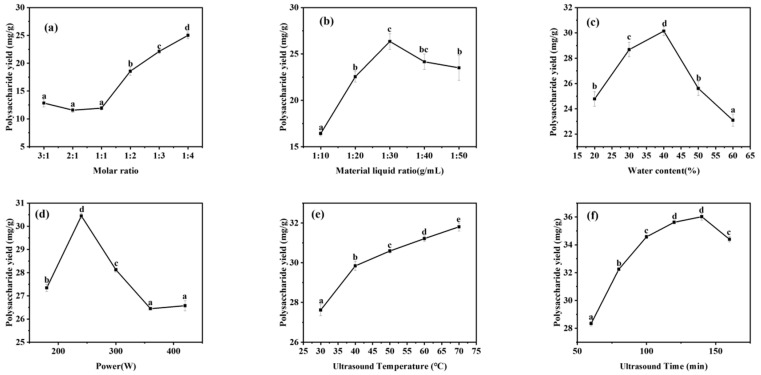
Single-factor assay: (**a**) Molar ratio; (**b**) Material liquid ratio; (**c**) Water content; (**d**) Power; (**e**) Ultrasound Temperature; (**f**) Ultrasound time. Note: values represent mean ± SD of three independent experiments; different letters represent significant differences (*p* < 0.05).

DESs generally exist in a homogeneous liquid state at ambient room temperature, and their melting point is somewhat lower than that of the ingredient chemistries that make up the system [49]. The melting point reflects the interaction of hydrogen bonds between the hydrogen bond acceptor and the hydrogen bond donor, and the molar ratio between the chemical components of DESs has a decisive effect on the melting point [50,51]. A significant factor affecting the extraction effect is the hydrogen bonding force between DESs; in other words, the molar ratio of DESs is significant for extracting polysaccharides, which is the first step in determining the extraction system. In this study, we mainly investigated the effect of the molar ratio (3:1, 2:1, 1:1, 1:2, 1:3, and 1:4) of DESs formulated with proline and malic acid under fixed conditions on the extraction effect of polysaccharides. It can be seen in Figure 4a that as the proline ratio increases, the extraction of polysaccharides becomes more effective. In 2012, María Francisco et al. [52] investigated the degradation of lignin, hemicellulose, and starch by various DESs. They found that in DESs prepared with malic acid as the hydrogen donor and proline as the hydrogen acceptor, the solubility of DESs for cellulose and starch also increased with an increasing molar ratio of proline. This may be because adding proline can enhance these DESs’ unique structure and increase the hydrogen bonding force. The degradation of lignin and cellulose increased, and it is speculated that the leaching of polysaccharides added more. Hence, the molar ratio experiment increased the number of extracted polysaccharides as the proportion of proline increased. The graph below shows that an increase in the molar ratios 1:3 and 1:4 decreases, and the ratio of malic acid/proline = 1:4 was chosen for thefollowing test in conjunction with economic factors.

### 2.5. The Effect of Material–Liquid Ratio on the Yield of ASPS

In this study, the optimization of DESs was carried out mainly for material–liquid ratios of 1:10, 1:20, 1:30, 1:40, and 1:50, and the best extraction effect of 26.35 ± 0.488 mg·g^−1^ can be seen in Figure 4b at a material–liquid ratio of 1:30. Between 1:10 and 1:30, the extraction effect was continuously enhanced. This finding may be attributed to the fact that with a gradual decrease in the material-to-liquid ratio, the contact area between the pulverized *A. senticosus* substance and the solvent was enlarged and full exposure was achieved, and the mass-transfer drive of the solvation was enhanced, which was conducive to the solubilization of the polysaccharide compounds. Under the condition of a material–liquid ratio greater than 1:30 g/mL, increasing the proportion of solvent leads to a decrease in the polysaccharide content, probably because diluting the solvent increases the ultrasonic energy consumption, which leads to ineffective cell breakdown. The effect of sonication of vaporization becomes less pronounced, thus reducing the amount of active material released. When the material–liquid ratio was 1:30 g/mL, the best possible extraction results were not only achieved, but the cost of the experiment was also saved. Thus, 1:30 g/mL was chosen for the following experiments.

### 2.6. The Effect of Water Content on the Yield of ASPS

In this study, the water content of DESs was optimized in the range of 20% to 60%, and it can be seen in Figure 4c that the extraction rate increased with an increase in water content, and the best extraction effect was achieved at 40% water content. The water content of the DES system has a significant effect on the leaching effect of polysaccharides, and DESs have a high viscosity, which is tens or even hundreds of times higher than the viscosity of water, so they are difficult to mix with the *A. senticosus* powder, and even the experimental operation is complex. DES polarity [53], to a certain extent, increases the extraction effect of DESs. However, with the addition of water at too high a level, the hydrogen bonds present in DESs will be reduced or even lost, changing the unique structure and, thus, leading to a significant decrease in the leaching rate of the target compounds. Therefore, DES6 with 40% water content was used to conduct the following study.

### 2.7. The Effect of Power on the Yield of ASPS

In this study, we mainly investigated the promotion effect of ultrasonic power between 160 W and 420 W on the extraction of polysaccharides using DESs. As shown in Figure 4d, the best extraction of polysaccharides was achieved when the ultrasonic power was 240 W. The leaching rate of polysaccharides kept increasing when the ultrasonic power increased from 160 W to 240 W. Nevertheless, the power was between 240 W and 420 W, and the extraction effect of polysaccharides decreased as the output tended to the equilibrium state. The higher the ultrasonic power, the faster the molecular vibration speed and the more pronounced the effect on the residue, promoting the extraction of polysaccharides in a low eutectic solvent [54]. However, the yield of polysaccharides decreased significantly under increasing ultrasonic power. A possible interpretation is that HF oscillations impact the formation of the internal network architecture of the DESs, and it is conjectured that too high a power also has an effect on the stabilization of the polysaccharide components, which reduces the ability to extract the polysaccharides from *A. senticosus*. Therefore, DES6 with an ultrasonic power of 240 W was selected for the following study.

### 2.8. The Effect of Temperature on the Yield of ASPS

This study mainly investigated the effect of temperatures between 30–70 °C on promoting polysaccharide extraction using DESs. It can be seen in Figure 4e that although the best extraction of polysaccharides was achieved when the sonication temperature was 70 °C, the effect of polysaccharide extraction was not significant when the temperature was 60 °C and 70 °C. The extraction rate of polysaccharides increased continuously during an increase in sonication temperature from 30 °C to 70 °C. Temperature is essential to DESs various physicochemical properties and functions [55]. As the temperature increases, the electrical conductivity of DESs increases and the surface tension decreases [56]. The increase in temperature accelerates the movement of molecules and decreases the viscosity of the solvent, thus increasing the compound dissolvability and the DESs’ exergy, which leads to an increase in the rate of mass transfer. For practical reasons, the sonication temperature of 60 °C was chosen for the following studies.

### 2.9. Investigation of the Effect of Time on the Extraction Effect of ASPS

This study mainly investigated the effect of time between 60–160 min on the promotion of polysaccharide extraction by DESs. From Figure 4f, it can be seen that the ultrasonication time between 60 min and 140 min increased the extraction of polysaccharides, and then the extraction amount stabilized and was not significant at 140 min. Pick-up time is the other significant argument that affects the ASPS pick-up rate. A potential explanation for these findings is that the dissolution rate of the target component increases with time, but the mass transfers made between the DES system and the *A. senticosus* powder equilibrated when the osmotic pressure of the full solution system reached equilibrium. In addition, a prohibitively long sonication time was associated with a significant increase in extraction cost. Finally, 140 min was chosen as the extraction time for further optimization.

### 2.10. PB Experimental Analysis

**Figure 5:**
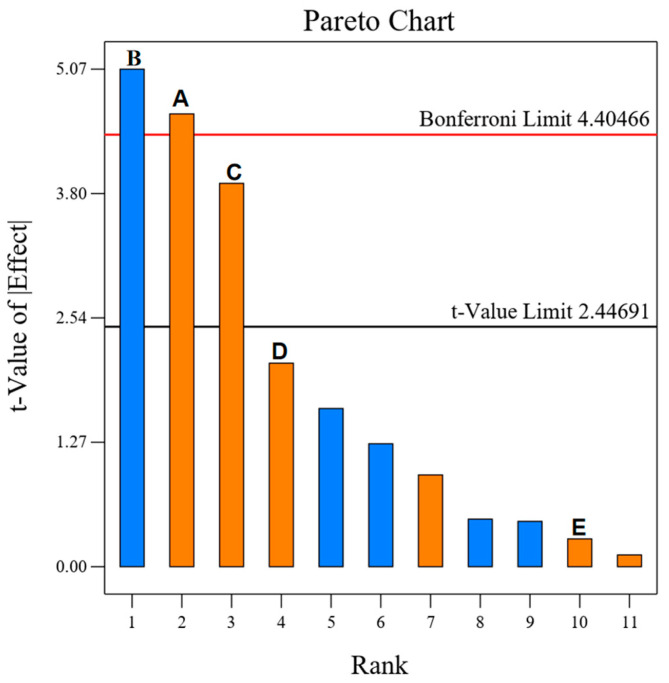
Pareto chart for the 5 factors of the PB experiment: (A) Material-iquid ratio; (B) Water content; (C) Ultrasonic time; (D) Ultasonic temperature; (E) Power.

As shown in Table 2 and Figure 5, it can be seen that according to the distribution of F and P corresponding to each influencing factor, it can be concluded that the degree of influence of the factors affecting the polysaccharide extraction rate of *A. senticosus* in descending order is as follows: B, A, C, D, and E. Among them, A, B, and C (*p* < 0.01) showed highly significant differences in the influence of DESs on the extraction of *A. senticosus* polysaccharides. From Figure 5, the Pareto chart, it is evident that the effect of the five factors on the polysaccharide extraction rate was in the order of B > A > C > D > E, with R^2^ = 0.9175 and adjusted R^2^ = 0.8487; the difference between the two is <0.2, and the model is reasonable. The Adeq Precision is 11.1025 > 4, indicating that the data are desirable and the model can be used as the basis for BBD response surface design. Therefore, the key factors A, B, and C were screened for the design calculation, optimization, and analysis of the BBD response surface.

### 2.11. Response Surface Method of Experimental Optimization Results and Analysis

#### 2.11.1. Response Surface Method of Optimization Test Results

Based on one-way and PB tests, based on the principle of Box–Behnken design, a three-factorial, three-level response surface test was designed to stabilize the ASPS extraction process, and the material–liquid ratio (A, g/mL), DES water content (B, %), and ultrasonic time (C, min) were obtained as the independent variables, and polysaccharide extraction rate (Y, mg·g^−1^) was obtained as the response variable in 17 sets of tests. The results of 17 sets of experiments are shown in Table 3. Quadratic regression was fitted to the data in Table 3 using Design Expert 13 software, and the regression model was obtained as:

Y = 33.94 + 0.1712A − 2.86B − 1.72C − 0.1900AB + 1.22AC + 4.4BC − 8.21A2 − 2.72B2 − 6.17C2 (1)

The data in Table 3 were subjected to ANOVA, and the results are shown in Table 4; the model F-value is 70.81 and *p* < 0.0001, which indicates that the model is highly statistically significant; the *p*-value of the out-of-fit term is 0.2017, which is not significant, which indicates that the equation is well fitted to the actual data, and the choice of this model to be used to design the analysis in optimum extraction criteria for ASPS is reasonable; and the value of the compound correlation coefficient R^2^ is 0.9891, which indicates that using this model can reflect the correlation between the three factors and polysaccharide extraction rate accurately enough. Adjusted R^2^ = 0.9752 and predicted R^2^ = 0.8812, indicating that the two are reasonably consistent, i.e., the difference between the two is <0.2. The Adeq Precision is 21.34 > 4, indicating that the model is reasonable and fits well. From the importance criterion, the primary (B and C), interaction (BC), and secondary (A^2^, B^2^, and C^2^) terms had a highly significant level of effect on the ASPS extraction rate (*p* < 0.01). Based on the F-value, the order of the magnitude of the effect of each factor on the percentage of total polysaccharides extracted was as follows: water content > sonication time > material–liquid ratio.

#### 2.11.2. Analysis of Response Surface Plots of the Interactions of the Factors

**Figure 6:**
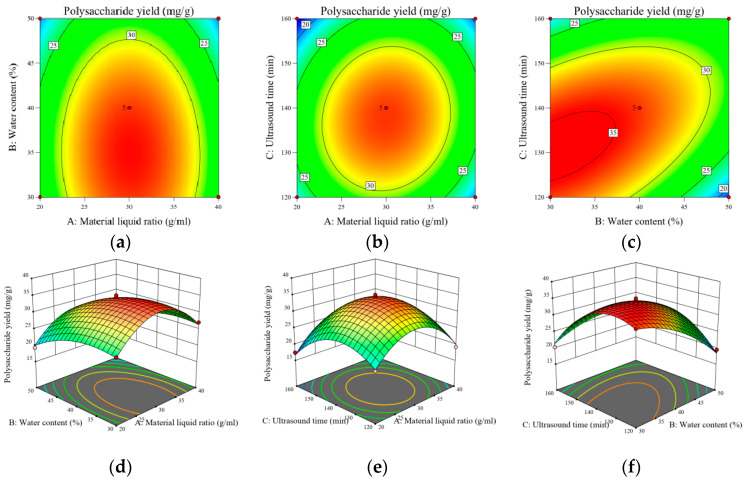
Three-dimensional response surface plots and contour plots (**a**–**f**) of the influence of interactions of various factors on the polysaccharide yield of *A. senticosus* polysaccharides.

The three-dimensional curve (down) and the corresponding contour (on top) derived from the quadratic multivariate fitting using the software can intuitively reflect the trend and degree of influence of the interaction between the factors on the polysaccharide yield (Y), as shown in Figure 6a–f. The surface inclination in the response surface is positively correlated with the interaction between the two factors. The surface has a larger inclination, which indicates that when the surface has a greater inclination, it indicates that the more sensitive the RES is to the change of the factor, i.e., the greater the effect of the factor on the polysaccharide yield, and conversely, the flatter the surface is, the lesser the effect of the factor is; the elliptical shape of the contour line indicates that the interaction between the two factors is significant, and if it is a circle, the significance is small.

As shown in Figure 6d, it can be seen that the surface plot is flat and its corresponding contour plot is approximately circular; the *A. senticosus* polysaccharide yield does not have a significant trend with changes in material–liquid ratio and water content, which indicates that the interaction between water content and material–liquid ratio does not have a significant effect on the *A. senticosus* polysaccharide yield. As shown in Figure 6e, the polysaccharide yield with increases in sonication time and material–liquid ratio firstly increases and then decreases, and the inclination of the surface is larger, and its contour is nearly elliptical, indicating that the interaction between the two has a greater effect on the *A. senticosus* polysaccharide yield. As shown in Figure 6f, it can be seen that the *A. senticosus* polysaccharide yield, with an increase in the water content and an increase in the sonication time, shows the tendency to firstly increase and then decrease, and the magnitude of the change is larger, and its contour is elliptical, indicating that the interaction of water content and sonication time has a greater effect on the polysaccharide yield. The interaction of water content and sonication time had a significant effect on the *A. senticosus* polysaccharide yield.

#### 2.11.3. Model Validation and Method Comparison

According to the prediction of model equation fitting, combined with the results of the above one-factor test, the optimal extraction conditions of polysaccharide were obtained as follows: material–liquid ratio = 31.068 g/mL, water content = 32.364%, extraction time = 129.119 min, and the predicted extraction rate of polysaccharides under this condition was 35.452 mg·g^−1^. Considering the practical operation, the optimum process was adjusted as follows: material–liquid ratio of 1:31 g/mL, water content of 32%, extraction time of 129 min, and three parallel extractions were carried out under these conditions. The results showed that the average extraction rate of ASPS was (34.931 ± 0.081) mg·g^−1^, which was close to the predicted value with a relative error of 1.469%. The results showed that the optimized process conditions of the method were reasonable and accurate. The same conditions were used for ultrasound-assisted aqueous extraction of *A. senticosus* polysaccharides, and the yield of *A. senticosus* polysaccharides extracted by DESs was 255.867% of that of the aqueous extraction with an average extraction rate of 13.652 ± 0.09 mg·g^−1^. In a 2018 study, Yong Chend et al. investigated the extraction of *Acanthopanax giraldii* using ultrasound assistance. They ultimately found that, under ideal circumstances, three repeats could provide the largest extraction quantity of 15.32 ± 0.37 mg·g^−1^ and that DESs could improve the output of *A. senticosus* polysaccharides by 128.1%. DESs not only show a good extraction effect but also have the advantages of green degradation and low time consumption and cost savings, so they have a better application prospect.

### 2.12. Kinetic Tests

#### 2.12.1. Secondary Kinetic Model

**Figure 7:**
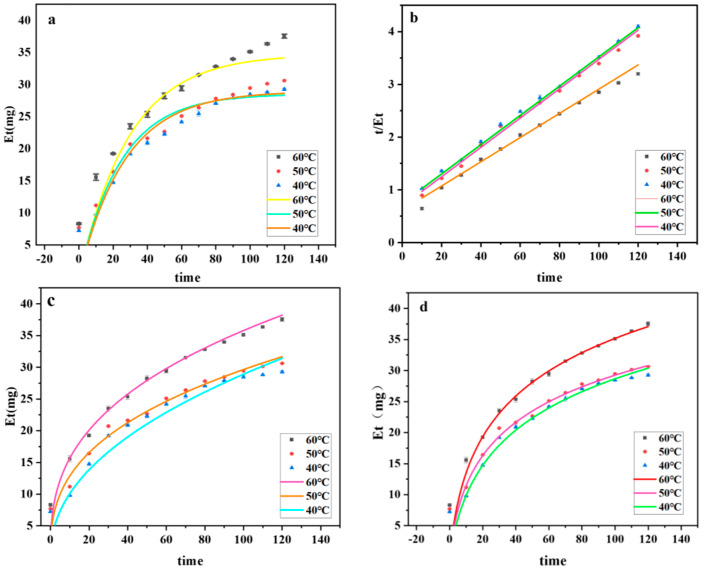
Dynamics fitting diagram. ((**a**): Secondary kinetic curves; (**b**): plot of “t/Et–leaching time”; (**c**): graph of a power law function; (**d**): Elovic curve plot).

As shown in Figure 7a, one can observe the fitted curves of ASPS extraction by DESs at different temperatures. It can be seen that the polysaccharide leaching rate proliferates at the beginning of extraction; when the extraction time exceeds 40 min, the growth rate of the polysaccharide leaching rate slows down. The initial rapid leaching of ASPS can be attributed to the driving force of water [57]. The secondary leaching model was plotted based on the secondary kinetic data, and Figure 7b shows the relationship between “t/Et-leaching time”. The agreement between the secondary leaching model and the experimental results confirms the hypothesis that there are two central phenomena in the leaching of ASPS: the initial stage (0–40 min), during which the maximum leaching of polysaccharides occurs, and the second stage (40–120 min), during which the leaching of polysaccharides depends mainly on external diffusion and is related to the number of residual polysaccharides in the matrix. Therefore, its extraction rate was slow [58].

Table 5 shows the secondary kinetic parameters of ASPS extraction by DESs at different temperatures, including the polysaccharide content Eeq at saturation, the extraction rate constant KB, the initial extraction rate h, and the coefficient of determination R^2^. From Table 5, it can be seen that the initial extraction rate h increases with increasing temperature in the range of 1.266 mg^2^·min^−1^ to 2.994 mg^2^·min^−1^; the temperature increases from the rate constant KB also increased from 0.00103 mg^2^·min^−1^ to 0.00158 mg^2^·min^−1^ during an increase in temperature from 40 °C to 60 °C. The rate constant of secondary leaching increased with increases in temperature.

#### 2.12.2. Power Law Model

The ASPS-PD yield in the figure is positively connected with the extraction temperature; a higher extraction temperature can increase the pace at which polysaccharides leach by reducing the density, viscosity, and surface tension of DESs. At all three temperatures, the power law kinetic model was satisfactorily fitted, and the expected and experimental data showed a high degree of agreement. The fitted model is more appropriate when the RMSE is smaller, as the Table 6 shows. At all three temperatures, the R^2^ is nearly equal to 1, and the RMSE of the results is smaller than 1. Since all of the results are smaller than 1, it is also possible to use the model to predict whether ultrasound-assisted DESs will be useful for extracting polysaccharides from *A. senticosus*.

#### 2.12.3. Elovich Kinetics Model

As shown in Figure 7, the ASPS-PD yield was positively correlated with the extraction temperature, and the Elovich kinetics model was successfully fitted at all three temperatures. The predictions of the Elovich kinetics model were in good agreement with the experimental results. As shown in Table 7, the initial extraction rate (E1) increased with the temperature at all three temperatures, which also indicated that the polysaccharide extraction rate increased with the temperature. R^2^ and RMSE also indicated the applicability of the model.

### 2.13. Reuse of DESs

DESs, as emerging extraction solvents with high extraction efficiency, can be recovered to realize their large-scale application. The polysaccharides were removed by alcohol precipitation after extraction by DESs, and the supernatant was taken and dried in a drying oven until the weight was no longer reduced. A new low eutectic solvent was reformulated for the second extraction. The second extraction was 83.4% of the first extraction, and the third was 46.24% of the first extraction. As the recovered DESs were reused more times, their ability to extract the target components decreased. The results suggest that DESs have good solvent integrity and can be recycled at least twice, resulting in more significant cost savings.

### 2.14. Morphological Characterization of A. senticosus Residues

**Figure 8:**
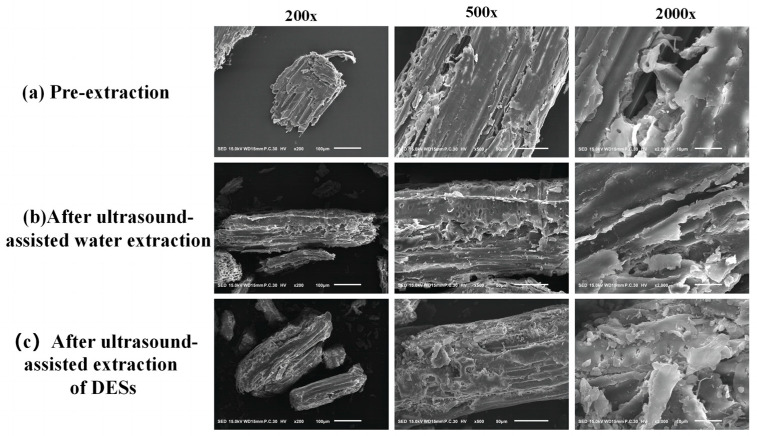
SEM images of *A. senticosus* residue after extraction.

In order to investigate whether the amount of polysaccharide release was related to the degree of destruction of plant cells in the extraction, morphological observations of three *A. senticosus* powders before extraction, after ultrasound-assisted water extraction, and after ultrasound-assisted DES extraction were carried out in this study at 200×, 500×, and 2000×, respectively. It can be seen that the cells of the pre-extraction powder (Figure 8a) were intact, the texture was clearly visible, and the cell walls were connected together and the surface was smooth without any fracture traces; in comparison, the other two extraction methods caused some damage to the surface of the *A. senticosus* powders, and the surfaces were rough, with wrinkles and fracture traces. The *A. senticosus* powder extracted by ultrasound-assisted DESs (Figure 8c) showed greater cellular rupture than that extracted by ultrasound-assisted water (Figure 8b), with obvious ruptures and collapses on the tissue surface and even tiny holes; the overall surface was rough and had many small irregular fragments. This suggests that DES caused damage to the cells; DES breaks down the cell wall and degrades the cellulose [23], so more target fractions are exposed and solubilized in the extract. From this, it can be determined that the degree of rupture of *A. senticosus* cells has a positive correlation with the effectiveness of ASPS extraction.

### 2.15. Comparison of Monosaccharide Composition, Molecular Weight, and Purity of ASPS-PD and ASPS-PW

As shown in the (Table 8), the molecular weight of ASPS-PD is lower than that of ASPS-PW, and the composition of monosaccharides is more varied than that of ASPS-PW and the purity of ASPS-PD is higher. And ASPS-PD has two more polysaccharides, xylose and mannose, than ASPS-PW. The results of the galacturonic assay test and the monosaccharide composition assay test were consistent, ASPS-PD possessed a higher galacturonic content. In addition, trace amounts of protein were detected in both PD and PW, suggesting that trace amounts of polysaccharide–protein complexes may be present in the extracts.

### 2.16. FT-IR Spectroscopy of Polysaccharides

**Figure 9:**
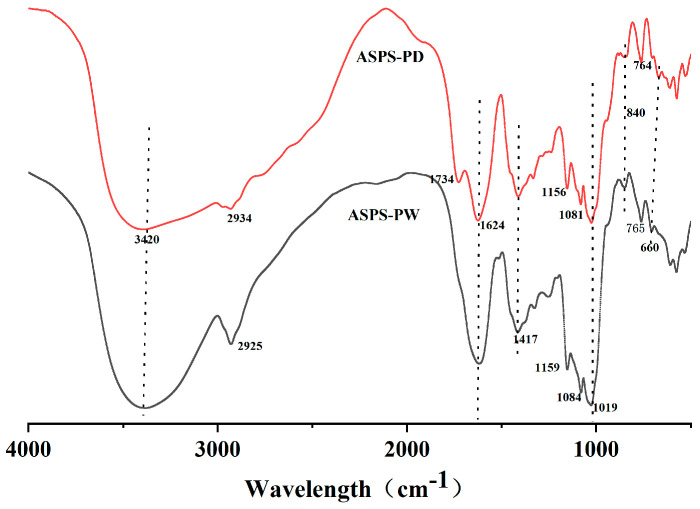
FT−IR spectra (polysaccharides).

The infrared spectrogram (Figure 9) shows the characteristic absorption peaks of polysaccharides; from the figure, it can be seen that there are broad and large diffraction peaks at 3420 cm^−1^ indicating the presence of hydroxyl (-OH) telescoping vibrations in the structure [59], and 2934 cm^−1^ and 2925 cm^−1^ are C–H telescoping vibrations indicating the presence of polysaccharides [60]. The characteristic absorption peaks of C=O in carboxylic acid esters are indicated at 734–1624 cm^−1^, suggesting the presence of glucuronic acid [61,62]. The small absorption peaks appearing at 1417 cm^−1^ are the variable-angle vibrational peaks of C–H, representing the presence of galactose [62]. Peaks at 1156 cm^−1^, 1081 cm^−1^, 1159 cm^−1^, and 1084 cm^−1^ are characteristic peaks of carbohydrates, which are mainly due to the overlapping of glycan ring vibrations. Peaks at 765 cm^−1^ and 764 cm^−1^ indicate the presence of a pyranose ring; and that at 840 cm^−1^ indicates the presence of an α-glycosidic linkage in the structure and the vibrational peaks appearing at and after 660 cm^−1^ are the fingerprint region; these peaks are typical of polysaccharides.

### 2.17. Ultraviolet (UV) Spectra

**Figure 10:**
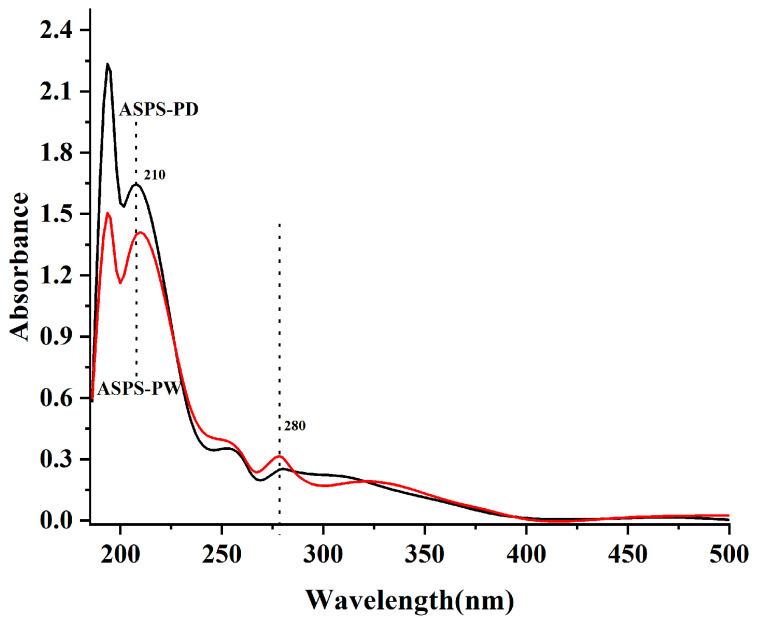
Ultraviolet spectrum (polysaccharides).

The scanned UV spectra (Figure 10) of the two polysaccharides showed that a weak absorption peak appeared at 280 nm for the two polysaccharides, ASPS-PW and ASPS-PD, indicating that the two polysaccharide fractions contain a small number of proteins, which is consistent with the results of the proteins measured in Table 8.

### 2.18. Comparison of ASPS-PD and ASPS-PW Oxidizing Activities

**Figure 11:**
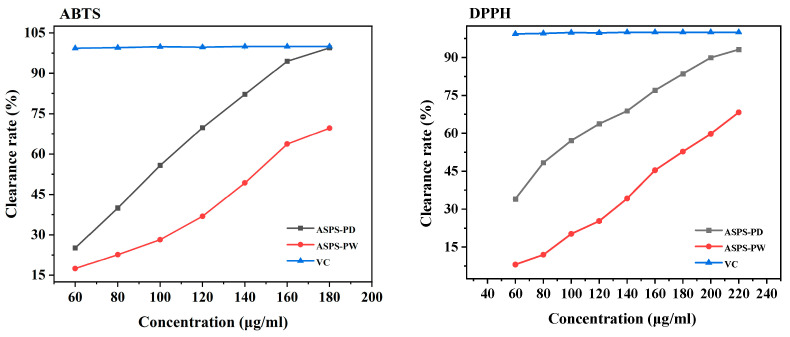
DPPH radical scavenging activities and ABTS radical scavenging activities.

As shown in Figure 11, the scavenging ability of ASPS-PD and ASPS-PW against DPPH and ABTS radicals, respectively, can be observed. It can be seen that as the concentration of the extract increased, its scavenging capacity for DPPH and ABTS free radicals was also more robust. Furthermore, both results showed that the antioxidant property of ASPS-PD was much higher than that of ASPS-PW. The IC_50_ of ASPS-PD in ABTS was 92.52 ± 3.169 μg/mL; the IC_50_ of ASPS-PW was 139.857 ± 4.589 μg/mL. The IC_50_ of ASPS-PD in DPPH was 89.861 ± 6.177 μg/mL; the IC_50_ of ASPS-PW was 175.943 ± 4.81 μg/mL. The antioxidant effect of polysaccharide extracts may be related to the different compositions of their monosaccharides, and it has been shown that the main active component in polysaccharides is glucuronic acid. The antioxidant results showed that ASPS-PW was more active, and the previous determination of the polysaccharide composition of ASPS-PD showed a higher glucuronic acid content than ASPS-PW, presenting results in agreement.

### 2.19. α-Amylase Inhibition Assay

**Figure 12:**
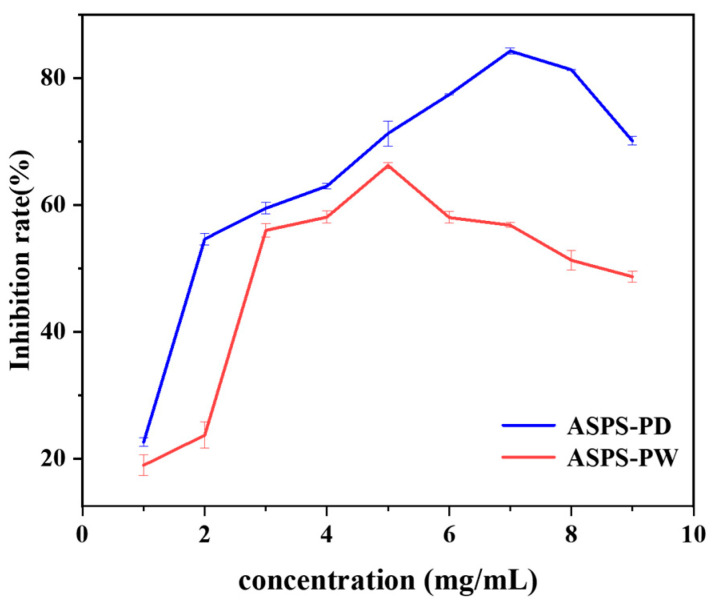
α-Amylase inhibition rate.

The picture below (Figure 12) shows that ASPS-PD’s ability to stop α-amylase from working quickly increases from 1 to 2 mg/mL. It then slowly increases from 2 to 7 mg/mL, reaching a maximum rate of 84.6 ± 0.467%. When the concentration of ASPS-PS is between 2 and 3 mg/mL, it quickly blocks α-amylase. Between 3 and 5 mg/mL, it slowly blocks them, reaching a peak of 66.2 ± 0.477%. This indicated that both ASPS-PD and ASPS-PW inhibited α-amylase activity, but the same dose of ASPS-PD showed better inhibition than ASPS-PW, which also explained the lower molecular weight of ASPS-PD [63]. Hwee-Feng Tan [63] determined that Momordica charantia polysaccharides can inhibit α-amylase up to 50% at 6.69 mg/mL, which is lower than the inhibition rate of ASPS-PD at the same concentration. The biological activity of polysaccharides is closely related to their monosaccharide composition. Hsu demonstrated that the anti-glycemic effect of polysaccharides is closely related to their own composition, and the higher the glucose and galactose content of polysaccharides, the better the anti-glycemic effect [64].

## 3. Materials and Methods

### 3.1. Materials and Reagents

*A. senticosus* Slices were purchased from Liaocheng Liming Pharmacy, ground into powder with a pulverizer (JYZ-B521, Joyoung Co., Ltd., Hangzhou, China), passed through a 150-mesh sieve, and stored at room temperature.

Lactic acid (ACS, ≥85%) and ethylene glycol (AR, 99.5%) were purchased from Aladdin Biochemical Technology Co., Ltd. in Shanghai, China. Choline chloride (AR, 98%), 1,4-butanediol (AR, 98%), L-choline chloride (AR, 98%), 1,4-butanediol (AR, 98%), L-malic acid (AR, 98%), L-proline (AR, 99%), urea (AR, 99%), and Betaine (AR, 98%) were purchased from Macklin Bio-chemical Technology Co., Ltd. in Shanghai, China. Oxalic acid (AR, 99.5%) was purchased from Fengchuan Chemical Reagent Technology Co., Ltd. in Tianjin, China. Anhydrous ethanol and sulfuric acid (AR, 95–98%) were purchased from the FEFC (Yantai, China) Co.

### 3.2. Preparation of DESs

Referring to the method of Shiyu Zhen [65], HBA and HBD were homogeneously mixed according to a certain molar ratio and stirred at 80 °C in a heat-collecting magnetic stirrer until complete dissolution, forming a homogeneous and stable colorless transparent liquid.

### 3.3. Choice of DESs

Choline chloride, betaine, 1,4-butanediol, propylene glycol, propionic acid, oxalic acid, L-malic acid, L-proline, lactic acid, and urea were made according to the method described in Section 2.2. The eight DESs were made in specific proportions (see Table 9). *A. senticosus* 0.5 g was accurately weighed and added to the configured DESs (30% water content) according to the material–liquid ratio of 1:20, sonicated at 240 W at 40 °C for 40 min, and the experiment was repeated three times. The extract was centrifuged at 8000 r/min for 10 min, and the supernatant was diluted ten times; anhydrous ethanol was added to 1 mL extract until the ethanol concentration reached 80%, and the content of polysaccharides was detected by the phenol–sulfuric acid method after alcoholic precipitation at 4 °C for 24 h. Additionally, this experiment also investigated a oxalic acid/choline chloride ratio = 2:1 with 30% water content, but it did not form a homogeneous liquid; no DESs were formed.

### 3.4. FTIR of DES

The spectral data of the three samples, L-proline, L-malic acid, and DES6, were recorded between 4000 and 400 cm^−1^ at room temperature using a Thermo Nicolet Is 10 instrument, and the functional groups of the three, as well as the relationship and dominance of DES6 and the other two, were deduced from the widths and sizes of the absorption peaks.

### 3.5. Selection of Extraction Method

DES6 with a water content of 30% was added to 0.5 g *A. senticosus* and used for extraction for 40 min in a shaker (speed: medium), in a water bath, and ultrasonically (240 W, 40 Hz) at 40 °C.

### 3.6. Effect of the Single Factor

We weighed 0.5 g *A. senticosus* and added DESs with changes to the following factors: molar ratio (3:1, 2:1, 1:1, 1:2, 1:3, 1:4); material–liquid ratio (1:10, 1:20, 1:30, 1:40, 1:50); water content (20%, 30%, 40%, 50%, 60%); power (120 W, 180 W, 240 W, 300 W, 360 W); temperature (30 °C, 40 °C, 50 °C, 60 °C, 70 °C); time (80 min, 100 min, 120 min, 140 min, 160 min).

### 3.7. Plackett–Burman Experiment (PB)

Based on the results of the single-factor experiments, five factors, material–liquid ratio (A), water content (B), ultrasound time (C), ultrasound temperature (D), and power (E), were selected for PB experiments, and the factors with more obvious correlation were selected to design response surface experiments. The experiment was divided into 12 groups, and the PB factor level table is shown in Table 10.

### 3.8. Response Surface Experiments

Apart from its strong prediction abilities, the Box–Behnken model may investigate the influence of two variables on extraction outcomes. Experiments on responsive surfaces are now used to optimize the extraction procedure [66,67]. The material–liquid ratio (X1), water content (X2), and time (X3) of the tri-factor response surface experiment were chosen after the findings of the PB and single-factor tests were analyzed. There are seventeen groups in the experiment, and Table 11 displays the response surface factor level.

### 3.9. Kinetics Models for Extraction

The present work investigated three kinetic models in order to ascertain the most suitable model for the experimental data. The models utilized in the kinetic study encompassed the power law model, the Elovich kinetics model, and the quasi-secondary kinetics model. In the context of kinetic modeling, the extraction temperature was manipulated at three different levels: 40 °C, 50 °C, and 60 °C. The extraction time was adjusted across a range of durations: 10, 20, 30, 40, 50, 60, 70, 80, 90, 100, 110, and 120 min. All other experimental variables were maintained at the optimal settings determined by the response surface analysis.

#### 3.9.1. Secondary Dynamics Models

According to the second-order adsorption kinetics equation [57]:

$$\frac{{\mathrm{dE}}_{t}}{d_{t}}=K_{B}\cdot {\left(E_{\mathrm{eq}}-E_{t}\right)}^{2}$$

where $K_{B}$ is the secondary reaction rate constant, $E_{\mathrm{eq}}$ is the reaction equilibrium extraction amount (μg), and $E_{t}$ is the extraction amount at time t (μg).

The above equation can be further simplified as${\;K}_{B}t=\frac{E_{t}}{{\left(E_{\mathrm{eq}}-E_{t}\right)}^{2}}$. The integral rate law for second-order extraction at t = 0 to t and${\;E}_{t}\;$= 0 to $E_{t}$ is

$$E_{t}=\frac{K_{B}\cdot t\cdot {E_{\mathrm{eq}}}^{2}}{1+K_{B}\cdot t\cdot K_{\mathrm{eq}}}$$

where h is the initial extraction rate at t close to 0.

$$h=K_{B}{E_{\mathrm{eq}}}^{2}$$

#### 3.9.2. Power Law Model

The power law model is a concise mathematical framework that can be employed to mimic the process of solid–liquid extraction, specifically focusing on the extraction of sizable organic molecules from botanical sources. The equation below can quantitatively represent the power law kinetic model [68]:

Et = Btn

where Et is the amount of polysaccharide leached (μg) at time t, n is the power law exponent (<1), and B is the constant associated with the extraction rate of the polysaccharide.

#### 3.9.3. Elovich Kinetics Model

The Elovich kinetics model can be used to describe the rate of a reaction as a function of time. The model has been used to describe the kinetics of vanillic acid extraction from pumpkin seeds [69]. The model equation is:

Et = E0 + E1 ln t

where Et is the extractable substance content (μg) at time t, E0 is the initial yield, and E1 is the initial extraction rate.

### 3.10. Reuse of DESs

The polysaccharide extract of DESs was alcoholically precipitated, and the supernatant was centrifuged and evaporated with alcohol and water in a drying oven until the weight of DESs remained constant. The dried liquid was taken, and water was added to prepare new DESs for polysaccharide extraction again, and the repeated utilization rate of DESs was calculated by repeating several times.

### 3.11. Scanning Electron Microscopy Assay

Scanning electron microscopy (SEM) is commonly used to observe the surface morphological structure of substances, and the three-dimensional spatial information of the sample surface was obtained by scanning the sample surface with an electron beam [70]. Appropriate amounts of the three *A. senticosus* powders before extraction, after ultrasound-assisted water extraction, and after ultrasound-assisted DESs extraction were taken, dried, thinly and uniformly adhered to a conductive adhesive, and mounted onto aluminum stakes, and after, the sprays of the gold plating were subjected to SEM at 200 X, 500 X, and 2000 X magnification under accelerating pressure of 10 kV.

### 3.12. Chemical Composition and Purity Analysis

Under the same conditions, polysaccharides were extracted from 5 g of *A. senticosus* powder by the DES extraction method and the hot water extraction method; regarding the crude polysaccharides obtained after precipitation with 80% ethanol, the precipitates were washed with anhydrous ethanol and methanol and then dissolved in distilled water and dried to obtain polysaccharides powders, named ASPS-PD and ASPS-PW. A total of 0.05 g of each powder was weighed and dissolved in 5 mL of water, and the contents of polysaccharides, reducing sugars, and proteins were determined. The results of polysaccharides, reducing sugars, proteins, and glucuronic acid are expressed as w%.

The purity of polysaccharides was calculated by the formula:

$$\mathrm{Purity}=\frac{W_{1}}{W}\%$$

where w_1_ is the weight of polysaccharides dissolving in water. W is the weight of dry polysaccharides.

### 3.13. FT-IR of Polysaccharides

One mg of dried sample was mixed with 150 mg of KBr powder, uniformly ground and pressed into tablets, then FTIR spectra were recorded from 400 to 4000 cm^−1^ using a Nicolet iS10 FTIR spectrometer.

### 3.14. Ultraviolet (UV) Spectra

The UV–visible spectra of the polysaccharide solutions were recorded in the 200–500 nm range using a UV–visible spectrophotometer (Nano Ready F-1100).

### 3.15. Molecular Weight Determination

HPGLC assessed the molecular weights of ASPS-PD and ASPS-PW. The samples and standards were weighed correctly and processed into a 5 mg/mL solution. The samples were centrifuged at 12,000 rpm for 10 min. The supernatant was filtered using a 0.22 μm micropore filter membrane after centrifugation. Finally, filtered samples were placed in 1.8 mL injection vials. A tandem gel column (8 × 300 mm) with the designation BRT105-104-102 was used in the experiment. A total of 0.05M NaCl was pumped at 0.6 mL/min as the mobile phase. We kept the column at 40 °C throughout the experiment. Samples of 25 μL were put into the column and detected using a RI-10A differential detector.

### 3.16. Carbohydrate Composition

The material was properly measured and placed in an ampoule at 5 mg. Two milliliters of 3M trifluoroacetic acid (TFA) was added to the ampoule. The combination hydrolyzed at 80 °C for 3 h. The hydrolyzed solution was pipetted into a tube and nitrogen blow-dried. After that, it was vortexed with 5 mL of water and 12,000 RPM centrifugation for 5 min. To analyze the supernatant, an ion chromatograph was used. The experiment utilized a 3 × 150 mm Dionex CarbopacTM PA20 column. This experiment employed three mobile phases: A, H2O; B, 15 mM NaOH; and C, a combination of 15 and 100 mM NaOAc. The mobile phase flow rate was 0.3 mL/min, with a 25 µL injection volume. The column temperature was 30 °C. The detector employed in this study was an electrochemical detector.

### 3.17. Antioxidant Activity of Polysaccharides

#### 3.17.1. DPPH Radical Scavenging Activity

The method of Shoib A. Bab [71] was modified slightly: 1 mL of sample solution at different concentrations (60, 80, 100, 120, 140, 160, 180, 200, 220 μg/mL) was added to 0.2 mmol/L of DPPH–ethanol solution, mixed well, and left to stand at 37 °C for 30 min, protected from light. The absorbance was measured at 517 nm by aspirating 200 μL into a 96-well plate and calculated according to the following equation:

$$\mathrm{DPHH}\;\mathrm{Free}\;\mathrm{radical}\;\mathrm{scavenging}\;\mathrm{rate}=\frac{A_{1}-\left(A_{2}-A_{3}\right)}{A_{1}}\times 100\%$$

where A_1_ is the absorbance of distilled water instead of the sample; A_2_ represents the absorbance of the sample; and A_3_ represents the absorbance of the DPPH–ethanol solution.

#### 3.17.2. ABTS Free Radical Scavenging Activity

The method of Ullah [72] was modified slightly: 7 mM ABTS was dissolved in potassium persulfate solution (2.5 mM) and left to stand for 14 h at room temperature in the dark to obtain the ABTS stock solution. The absorbance at 734 nm was 0.700 ± 0.020 by diluting the ABTS stock solution with ethanol. A total of 0.2 mL of the sample solution at different concentrations (60, 80, 100, 120, 140, 160, 180, 200 μg/mL) was mixed with 0.8 mL of ABTS, and the reaction was carried out for 6 min, then μL was taken in a 96-well plate, and the absorbance was measured at 734 nm. The absorbance was measured at 734 nm and calculated according to the following equation:

$$\mathrm{ABTS}\;\mathrm{formula}\;\mathrm{free}\;\mathrm{radical}\;\mathrm{scavenging}\;\mathrm{rate}=\frac{B_{1}-\left(B_{2}-B_{3}\right)}{B_{1}}\times 100\%$$

where B_1_ represents the absorbance of the sample replaced by distilled water; B_2_ represents the absorbance of the sample; and B_3_ represents the absorbance of the ABTS.

### 3.18. α-Amylase Inhibition Assay

A slightly modified approach was used to determine the inhibition of α-amylase [73]. The following reagents were added: 1.0 mL of polysaccharide solution at various concentrations (1.0, 2.0, 3.0, 4.0, 5.0, 6.0, 8.0, and 9.0 mg/mL), 0.3 mL of α-amylase solution (enzyme activity: 5 U/mL), and 0.4 mL of 1% soluble starch solution. The mixture was heated in a water bath at 37 degrees for 5 min. After that, 2 mL of DNS reagent was added, and the reaction was allowed to boil for 10 min. The reaction was then allowed to cool to room temperature before the absorbance was measured at 540 nm.

$$\alpha \text{-}\mathrm{Amylase}\;\mathrm{inhibition}\;\mathrm{rate}\;(\%)=\frac{A_{1}-A_{3}}{A_{2}-A_{4}}\times 100\%$$

where A_1_ is the absorbance of sample solution + enzyme solution; A_2_ is the absorbance of PBS punch + enzyme solution; A_3_ is the absorbance of sample solution + PBS buffer; and A_4_ is the absorbance of PBS buffer.

### 3.19. Data Analysis

For the Box–Behnken experimental design, Design-Expert 13 software was used. Origin 2021 was used for plotting, and SPSS statistical software (version 13.0) was used to analyze the data. The data were shown as mean ± SD, and one-way ANOVA was used for testing.

## 4. Conclusions

This study established an ecologically friendly extraction system to extract polysaccharides from *A. senticosus* efficiently. Because the polarity, composition, and water content of DESs can affect the extraction efficiency of target components, the appropriate composition and ratio of DESs are extremely important in improving the extraction rate of target components. It was verified that the best extraction was achieved by a deep eutectic system consisting of L-malic acid and L-proline at a molar ratio of 1:4, and the extraction of ASPS was assisted by an ultrasonic power condition of 240 W. Then, specific extraction process parameters (material–liquid ratio of 31.068 g/mL, water content of 32.364%, and extraction time of 129.119 min) were quickly and accurately screened by a one-way test and response surface methodology. The results showed that the extraction rate was superior to that of the hot water extraction method under the optimized conditions. Subsequently, a kinetic study was carried out for the three kinetic models, and based on the results, it was found that the secondary kinetics model is more suitable for different temperatures according to the extraction of ASPS; the R^2^ is up to 0.99 or more. The surface structure of the *A. senticosus* powder obtained from both extraction methods was looser, with a multi-reticulated sheet-like structure. However, the surface of the samples extracted with DES was rougher, and its fibrous organization was destroyed to a greater extent, even in fragments. Meanwhile, the polysaccharides in DESs can be extracted by alcohol precipitation and reused. The extracted polysaccharides have a greater monosaccharide composition and lower molecular weight compared to the water-extracted polysaccharides and have a better anti-glycation effect. Therefore, this study demonstrates that DESs can be used as a promising, cost-effective, and sustainable natural product extraction medium for extracting polysaccharides from TCM. It lays a solid basis for their practical use in medical, food, and other fields.

However, this study still has some limitations. Firstly, certain conditions need to be met for the preparation of low eutectic solvents, such as suitable temperature and uniform stirring, which makes it difficult to achieve large-scale production; secondly, the stability of the extraction rate of the target components by the DES method is not high; and lastly, even though the extraction method is simple to operate, further optimization of the conditions and steps of the extraction process is required if it is to be commercialized.

## Figures and Tables

**Table 1:** Ultrasound-assisted extraction of polysaccharides from different plants using different types of deep eutectic solvent.

Raw Material	Types/Molar Ratio	Extraction Mean	Extraction Temperature	Extraction Time	Yield	Ref.
Dioscorea opposita Thunb	Chcl–1,4-Buta = 4:1	UAE	94 °C	44.7 min	15.98 ± 0.15%	[42]
Abalone viscera	Chcl–Eg = 1:1	UAE	73 °C	40.0 min	16.84 ± 1.32%	[43]
Anji White Tea	Chcl–1,6-CH = 1:2	UAE	-	40.0 min	19.18%	[44]
Indocalamus tessellatus leaves	Chcl–Ma = 1:4	UAE	63.5 °C	60.0 min	0.68%	[45]
Astragalus membranaceus	Chcl–Ur = 1:1	-	60 °C	90.0 min	141.11 mg·g^−1^	[46]
Sweet Tea Leaves	Chcl–Eg = 1:2	MAE	-	10.0 min	4.16% ± 0.09%	[47]

Chcl: choline chloride; Eg: ethylene glycol; 1,4-Buta: 1,4-butanediol; 1,6-CH: 1,6-hexanediol; Ma: malonic acid; Ur: urea.

**Table 2:** Effect size and significance analysis of each factor.

Source	Sum of Squares	df	Mean Square	F-Value	*p*-Value	
Model	371.37	5	74.27	13.34	0.0034	significant
Material–liquid ratio (A): g/mL	118.65	1	118.65	21.31	0.0036	***
Water content (B): %	143.28	1	143.28	25.74	0.0023	***
Ultrasound time (C): min	85.04	1	85.04	15.27	0.0079	***
Ultrasound temperature (D): °C	23.96	1	23.96	4.30	0.0834	
Power (E): W	0.4428	1	0.4428	0.0795	0.7874	
Residual	33.40	6	5.57			
Cor Total	404.78	11				
R^2^	0.9175					
Adjusted R^2^	0.8487					
Predicted R^2^	0.6699					
Adeq Precision	11.1025					

*** indicates difference is highly significant (*p* < 0.01).

**Table 3:** Response surface optimization of investigated variables using DES6 as extraction solvent.

Run	A Material–Liquid Ratio (g/mL)	B Water Content (%)	C Ultrasound Time (min)	Polysaccharide Yield (mg/g)
1	30	40	140	33.9371
2	20	50	140	19.31
3	40	40	120	19.23
4	30	40	140	32.85
5	30	50	120	19.516
6	30	50	160	24.721
7	40	50	140	19.68
8	40	40	160	19.82
9	20	30	140	25.95
10	30	40	140	34
11	30	30	120	34.1625
12	30	30	160	20.33
13	30	40	140	35.1
14	20	40	160	17.45
15	30	40	140	34.542
16	40	30	140	27.08
17	20	40	120	21.73

**Table 4:** ANOVA of the regression model for extraction efficiency of polysaccharides.

Source	Sum of Squares	df	Mean Square	F-Value	*p*-Value	Significance
Model	725.02	9	80.56	70.81	<0.0001	significant
A—Material–liquid ratio	0.2346	1	0.2346	0.2062	0.6635	
B—Water content	73.78	1	73.78	64.86	<0.0001	***
C—Ultrasound time	18.97	1	18.97	16.67	0.0047	**
AB	0.1444	1	0.1444	0.1269	0.7321	
AC	5.93	1	5.93	5.21	0.0564	
BC	90.61	1	90.61	79.65	<0.0001	***
A^2^	276.45	1	276.45	243.01	<0.0001	***
B^2^	37.34	1	37.34	32.82	0.0007	**
C^2^	173.84	1	173.84	152.81	<0.0001	***
Residual	7.96	7	1.14			
Lack of Fit	5.17	3	1.72	2.47	0.2017	not significant
Pure Error	2.79	4	0.6983			
Cor Total	732.98	16				
R^2^	0.9891					
Adjusted R^2^	0.9752					
Predicted R^2^	0.8812					
Adeq Precision	213400					

***: indicates highly significant difference (*p* < 0.01); **: indicates a significant difference (*p* < 0.001).

**Table 5:** Secondary kinetic parameters at different temperatures.

Temperature/°C	Eeq/mg	kB/mg·min^−1^	h	R^2^
40 °C	35.06	0.00103	1.266	0.99966
50 °C	37.90	0.00112	1.608	0.99315
60 °C	43.57	0.00158	2.994	0.99815

**Table 6:** Power law function parameters at different temperatures.

Temperature (°C)	B	n	R^2^	RMSE
40 °C	3.52	0.46	0.96	0.69
50 °C	5.59	0.36	0.87	0.80
60 °C	6.86	0.36	0.92	0.48

**Table 7:** Elovich kinetics model parameters at different temperatures.

Temperature (°C)	E0	E1	R^2^	RMSE
40 °C	0.10	1.70	0.97	0.57
50 °C	0.11	2.37	0.86	0.49
60 °C	0.09	2.70	0.927	0.66

**Table 8:** Physico-chemical properties of ASPS-PD and ASPS-PW.

Physicochemical Properties	ASPS-PD	ASPS-PW
Reducing sugar (w%) a	0.105 ± 0.006 *	0.156 ± 0.012 *
Glucuronic acid (w%) a	34.23 ± 0.42 *	28.3 ± 0.35 *
Total proteins (w%) a	4.46 ± 0.1 *	2.55 ± 0.08 *
purity (w%) a	55 ± 0.47 *	43.09 ± 0.28 *
Molecular weights (Mw, kDa)	2.981	9.984
Monosaccharide composition (mol%) b		
Rhamnose	0.022	0.029
Arabinose	0.058	0.089
Glucose	0.531	0.679
Galactose	0.183	0.136
Xylose	0.029	- c
Mannose	0.046	- c
Galacturonic	0.130	0.05

Data are represented by mean ± SD, which is the mean of three replicates. a: weight percentage. b: molar ratio. c: not detected. *: *p* < 0.01.

**Table 9:** DESs type.

	DES Type	Molar Ratio	Water Content	pH
DESs1	Chcl–Ur	2:1	30%	8.64
DESs2	Beta–Eg	1:3		9.32
DESs3	Chcl–1,4-Buta	1:2		5.06
DESs4	Beta–1,4-Buta	1:3		7.58
DESs5	Chcl–La	2:3		1.55
DESs6	L-Ma–L-Pro	1:3		4.4
DESs7	Chcl–L-Ma	3:2		3.28
DESs8	L-Pro–La	1:2		3.01

Note: Chcl: choline chloride; Ur: urea; Beta: betaine; Eg: ethylene glycol; 1,4-Buta: 1,4-butanediol; La: lactic acid; L-Ma: L-malic acid; L-Pro: L-proline.

**Table 10:** Factors and levels of Plackett–Burman design.

Factor	Level	
	−1	1
Material liquid ratio (A): (g/mL)	1:20	1:40
Water content (B): %	30	50
Ultrasound Time (C): min	120	160
Ultrasound Temperature (D): °C	50	70
Power (E): W	180	300

**Table 11:** Box–Behnken experimental factor level.

Factor	Level		
	−1	0	+1
Material–liquid ratio (A): g/mL	1:20	1:30	1:40
Water content (B): %	30	40	50
Ultrasound Time (C): min	120	140	160

## Data Availability

Data are contained within the article.
